# Cost Efficacy of Rapid Whole Genome Sequencing in the Pediatric Intensive Care Unit

**DOI:** 10.3389/fped.2021.809536

**Published:** 2022-01-24

**Authors:** Erica Sanford Kobayashi, Bryce Waldman, Branden M. Engorn, Katherine Perofsky, Erika Allred, Benjamin Briggs, Chelsea Gatcliffe, Nanda Ramchandar, Jeffrey J. Gold, Ami Doshi, Elizabeth G. Ingulli, Courtney D. Thornburg, Wendy Benson, Lauge Farnaes, Shimul Chowdhury, Seema Rego, Charlotte Hobbs, Stephen F. Kingsmore, David P. Dimmock, Nicole G. Coufal

**Affiliations:** ^1^Department of Pediatrics, Cedars-Sinai Medical Center, Los Angeles, CA, United States; ^2^Rady Children's Institute for Genomic Medicine, San Diego, CA, United States; ^3^Rady Children's Hospital San Diego, San Diego, CA, United States; ^4^Naval Medical Center San Diego, San Diego, CA, United States; ^5^McGovern Medical School, University of Texas Health Science Center at Houston, Houston, TX, United States; ^6^Department of Neuroscience, University of California, San Diego, San Diego, CA, United States; ^7^Department of Pediatrics, University of California, San Diego, San Diego, CA, United States; ^8^Department of Infectious Disease, University of California, San Diego, San Diego, CA, United States

**Keywords:** genomic sequencing, rapid whole genome sequencing, pediatric intensive care, critical care, cost analysis, health economics, quality adjusted life year (QALY)

## Abstract

The diagnostic and clinical utility of rapid whole genome sequencing (rWGS) for critically ill children in the intensive care unit (ICU) has been substantiated by multiple studies, but comprehensive cost-effectiveness evaluation of rWGS in the ICU outside of the neonatal age group is lacking. In this study, we examined cost data retrospectively for a cohort of 38 children in a regional pediatric ICU (PICU) who received rWGS. We identified seven of 17 patients who received molecular diagnoses by rWGS and had resultant changes in clinical management with sufficient clarity to permit cost and quality adjusted life years (QALY) modeling. Cost of PICU care was estimated to be reduced by $184,846 and a total of 12.1 QALYs were gained among these seven patients. The total cost of rWGS for patients and families for the entire cohort (38 probands) was $239,400. Thus, the net cost of rWGS was $54,554, representing $4,509 per QALY gained. This quantitative, retrospective examination of healthcare utilization associated with rWGS-informed medicine interventions in the PICU revealed approximately one-third of a QALY gained per patient tested at a cost per QALY that was approximately one-tenth of that typically sought for cost-effective new medical interventions. This evidence suggests that performance of rWGS as a first-tier test in selected PICU children with diseases of unknown etiology is associated with acceptable cost-per-QALY gained.

## Introduction

Rapid whole genomic sequencing (rWGS) is transforming the diagnosis of single locus genetic disease among inpatient children. Recent technological advances enable return of results in less than one day, which enables timely provision of optimal care broadly for critically ill patients, effecting immediate changes in the trajectory of their clinical management (1–5). Mounting evidence supports the diagnostic and clinical utility of rWGS and was further substantiated by a recent meta-analysis (6). Though earlier studies were focused primarily on infants in the neonatal intensive care unit (NICU), recent studies examining children outside of the neonatal period in the pediatric intensive care unit (PICU) have yielded similar diagnostic and clinical utility rates (5, 7, 8). Our group recently published a retrospective cohort study of 38 critically ill children in the PICU and found that rWGS resulted in a molecular diagnosis in 17 of 38 children (45%) (5). Seventy-six percent of diagnoses affected clinical management of the patient (5).

Despite increasingly clear-cut clinical evidence supporting use of rWGS, several barriers have precluded its widespread implementation. One of the most significant is the ongoing need for prior authorization for reimbursement. To achieve rWGS coverage policy determinations, payors frequently raise concern related to the associated cost. To buttress the proposition that timely diagnosis may decrease overall costs by optimizing clinical management, several studies have recently demonstrated cost-effectiveness for early utilization of rWGS or rapid whole exome sequencing (rWES) compared to traditional diagnostic investigations (9–12). In 2018, Stark et al. estimated the cost savings in a group of 21 critically ill children who received a diagnosis by rWES to be AU$543,178 (US$424,101), due to avoidance of planned tests and procedures and reduced length of stay (13). Schofield et al. expanded the cost analysis of a cohort of 80 infants in Australia to include increased projected quality life-years (QALYs) attributable to early molecular diagnosis as well as the economic effects of cascade testing in first-degree relatives (14). Each of these models resulted in incremental cost savings, lending further credence to the assertion that early deployment of rWGS in suspected single locus genetic disorders is increasingly cost-effective (14). Though compelling, the aforementioned precision medicine health economics analyses were performed outside of the United States, specifically in single-payer systems such as Australia (15) which limits its applicability to the complex and fractured United States health care system (12, 14–17).

Recent analyses of critically ill infants diagnosed by rWGS have found associated reductions in hospital length of stay and significant net cost savings (4, 9, 17). However, in nearly all cases, the infants studied were from the NICU (4, 9, 17). Though these studies demonstrated the cost-effectiveness of genomic sequencing for acutely ill infants, a fiscal examination exclusive to the PICU (where the average age is much older) has not been done.

Additionally, only a modest number of groups have endeavored to include QALYs as the outcome measure in their economic analyses, as recommended by major health technology assessment agencies when performing a cost-utility analysis (4, 14, 18, 19). The QALY is the preferred metric because it combines two different benefits attributed to a particular intervention- longevity and quality of life- into a single number that can be compared between alternative interventions (20). The monetary value of a QALY (cost per QALY) is a marker of value used to establish the comparative value of different healthcare interventions. A QALY is used to optimally direct resources (21–23). Historically, in the US the QALY benchmark for a clinical intervention that is worth the investment has been $50,000, though it has not been adjusted for inflation and suggestions have been made recently that this number should be increased (22, 24). Thus, inclusion of QALYs is imperative to any cost-utility analysis because health outcomes and improvement of said outcomes is arguably the driving goal behind the allocation of limited healthcare resources.

In a world of finite health care resources, studies are urgently needed to determine whether rWGS in the PICU setting is an efficient use of limited health care resources, and thereby advise policy development and resource allocation. We endeavored to measure the financial and clinical impact of rWGS on acutely ill children using established measures of comparative effectiveness.

## Results

### Study Population

This was an economic analysis of a previously published cohort of critically ill children who received rWGS. The patients' clinical characteristics have been previously described (5). Briefly, this cohort included 38 children ranging in age from 4 months to 17 years (mean 5.73 years) (5). Of the 38 study participants, 17 received a molecular diagnosis (45%) and 13 of the 17 (76%) had an associated change in clinical management (5). Aside from the increased costs of diagnostic testing, two patients (6180 and 6207) were identified to have an increased cost of care attributable to rWGS testing (related to additional intravenous immunoglobulin administration and earlier initiation of Factor XIII replacement). Detailed cost and QALY modeling were performed for eight of the 13 patients who had a change in clinical management (Table 1). Quantitative modeling was not possible in the other five patients with changes in management following rWGS-based diagnoses (Supplementary Table 1). None of these five cases were thought to likely have a net increase in the estimated cost of care. They included patients 6031, 6118, and 7002 in whom there was a lack of substantial literature to precisely predict the morbidity risk avoided by the intervention, and patients 6183 and 7039 for whom there was insufficient documentation to substantiate specific clinical management changes that ensued following palliative care consultation.

**Table 1:** Precision medicine interventions in eight of 13 patients who received molecular diagnoses and had resultant changes in clinical care.

**Patient**	**Gene**	**Diagnosis name**	**Intervention modeled**	**Delphi panel consensus (Y/N)**	**Cost savings/ costs incurred**	**QALY savings**
6007	*PCDH19*	Early infantile epileptic encephalopathy	Pulse steroids instead of ICU transfer for midazolam infusion	Y	$9,795	-
6052	*TANGO2*	Metabolic encephalomyopathic crises, recurrent, with rhabdomyolysis, cardiac arrhythmias, and neurodegeneration (MECRCN)	Carries letter describing diagnosis/treatment recommendations. Subsequent acute encephalopathic episode improved secondary to recommendations	N	n/a	n/a
6147	*TRNT1*	Sideroblastic anemia with B cell immunodeficiency, periodic fevers, and developmental delay (SIFD)	Change in family's goals of care avoided one hospitalization, skin biopsy, and EGD/intestinal biopsies	Y	$74,556	-
6153	*AIRE*	Autoimmune polyendocrinopathy syndrome, type I	Vaccination for encapsulated organisms decreased risk of mortality	Y	-	0.12
6159	*COL4A4*	Thin basement membrane nephropathy/ Alport syndrome	Avoided a renal biopsy	Y	$8,108	-
6180	*BTK*	Agammaglobulinemia, X-linked	Received 6 additional doses of IVIG	Y	-$9,856 (incurred cost)	-
6193	*NALCN*	Congenital contractures of the limbs and face, hypotonia, developmental delay (CLIFAHDD)	Transitioned to home care with non-invasive positive pressure ventilation on hospice instead of remaining in hospital	Y	$134,538	-
6207	*F13A1*	Factor XIIIA deficiency	Decreased risk of repeat CNS bleed and associated mortality and neurologic complication by initiating prophylactic Factor XIII replacement	Y	-$32,295 (incurred cost)	11.98
Total:					$184,846	12.1

*ICU, intensive care unit; EGD, esophagogastroduodenoscopy; IVIG, intravenous immunoglobulin; CNS, central nervous system*.

### Cases Modeled (Reached Delphi Consensus)

A Delphi consensus was reached in 7 of 8 subjects receiving a molecular diagnosis resulting in a previously reported clinical management change (Table 1). Detailed counterfactual trajectories and the questions posed (as presented to the Delphi panelists) are available in Supplementary Material. The seven counterfactuals to reach consensus are briefly described herein. For the eighth patient, 6052, who was diagnosed with a homozygous recessive variant in *TANGO2* (OMIM#616878; Metabolic encephalomyopathic crises, recurrent, with rhabdomyolysis, cardiac arrhythmias, and neurodegeneration) the Delphi panel failed to reach consensus on whether a molecular diagnosis likely reduced the length of hospital stay for a metabolic crisis.

### Cases With Cost Savings and/or QALY Savings

For Patient 6007, rWGS identified a deletion in *PCHD19* (OMIM#300088; Developmental and epileptic encephalopathy 9) resulting in pulse dose methylprednisolone for refractory seizures and avoidance of PICU care for a midazolam infusion (Table 1). For Patient 6147, diagnosis of homozygous variants in *TRNT1* (OMIM#616084; Sideroblastic anemia with B-cell immunodeficiency, periodic fevers, and developmental delay) led to palliative care consultation and a change in ‘code status' to ‘Allow Natural Death'. The family chose to decline diagnostic procedures and an admission for treatment of sepsis, in line with their shifted goals of care. Patient 6153 was diagnosed with compound heterozygous variants in *AIRE* (OMIM#240300; Autoimmune polyendocrinopathy syndrome, type I) and was subsequently advised to receive 23-valent pneumococcal and meningococcal vaccination due to risk of development of functional asplenism, decreasing her long-term risk of morbidity and mortality due to infection with these encapsulated organisms. A renal biopsy had been recommended for patient 6159 during her ICU admission as part of her diagnostic workup for acute renal failure, but once rWGS revealed a likely pathogenic variant in *COL4A4*, associated with Alport syndrome/thin basement membrane nephropathy (OMIM#141200; thin-basement-membrane nephropathy), the nephrologist canceled plans for the biopsy. Though *COL4A4* is listed on commercial gene panels for nephrotic syndrome, the turnaround time is 4 weeks and results would not have returned prior to the planned biopsy. Patient 6193 was admitted for respiratory failure and rWGS revealed a *de novo*, likely pathogenic variant in *NALCN* (OMIM#616266; Congenital contractures of the limbs and face, hypotonia, and developmental delay), which resulted in a palliative care consult and facilitated the transition to home non-invasive positive pressure ventilation (NPPV), as opposed to ongoing hospitalization while awaiting symptom resolution.

### Cases With Incurred Costs

For Patient 6180, admitted to the PICU for pseudomonal septic shock, the rWGS diagnosis of a *de novo*, pathogenic variant in *BTK* (OMIM#300755; Agammaglobulinemia, X-linked 1) led to administration of additional doses of intravenous immunoglobulin (IVIG) (Table 1). Costs associated with the extra IVIG administration were considered as costs incurred as a result of the genomic diagnosis. Cost savings of preventing further disease morbidity from delayed diagnosis of primary immunodeficiency and unmitigated sepsis were not calculated. Patient 6207 was an 8-month-old who presented with right arm paralysis and was found to have a large intraparenchymal hematoma. rWGS identified homozygous variants in *F13A1* (OMIM#613225; Factor XIIIA deficiency), and he was immediately started on prophylaxis with recombinant coagulation FXIII A-subunit, decreasing his risk for another central nervous system (CNS) bleed and the associated potential neurologic complications and mortality. Costs associated with the earlier administration for Factor XIII were considered as costs incurred as a result of the genomic diagnosis.

### Impact of rWGS-Associated Precision Medicine on Healthcare Utilization

The total cost for clinical investigations, interventions, procedures, medications, and inpatient room stays, as well as QALYs, when applicable, are presented in Table 1 for the seven patients and Delphi questions that reached consensus. Estimated cumulative savings due to rWGS were $184,846. Over 70% of estimated savings derived from reduced or avoided hospital length of stay. The remainder was due to avoided professional fees and major procedures (Table 1). The estimated charge for each avoided or incurred intervention can be found in Supplementary Table 2.

### QALY Modeling

QALY modeling was performed and QALY gain was identified for the two patients with Delphi consensus. The total estimated benefit from rWGS in these two children was 12.1 QALYs saved (patients 6153 and 6207). The greatest benefit was in patient 6207, who was diagnosed with severe Factor XIII deficiency (11.98 QALYs saved). Without treatment, his risk of dying if he were to have a second central nervous system (CNS) bleed would be 12%, with the mean age of death being 1.5 ± 2 years (26–29). QALYs saved by avoiding this risk of death amounted to 9.08 (Supplementary Table 3). Additionally, of patients who survive a subsequent CNS bleed, 72.6% have neurologic complications (28). Prophylaxis with Factor XIII replacement resulted in a gain of 2.9 QALYs due to avoided neurologic disability (Supplementary Table 3).

### Cost of rWGS

An estimated cost of $7,400 was applied per trio rWGS performed. This is an average of total costs from previous cases sequenced at Rady Children's Institute for Genomic Medicine (RCIGM) and includes the cost sequencing and interpretation. Total cost of rWGS for the entire cohort of 38 probands and their families was $239,400 (Supplementary Table 4).

### Cost Effectiveness Analysis

**Figure 1:**
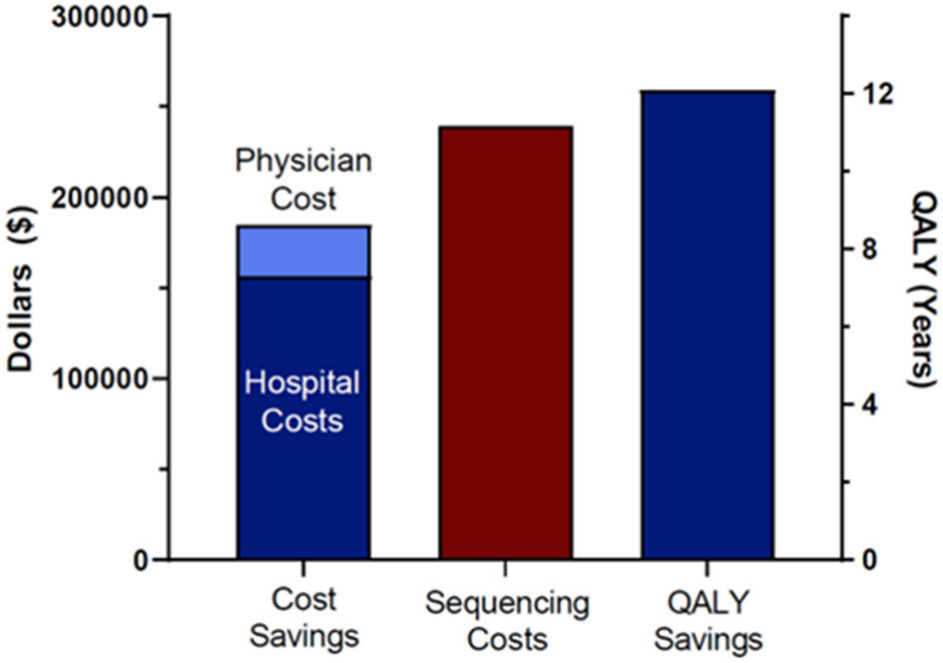
Cost savings, sequencing costs, and QALY saving. Sequencing costs included all 38 patients in the original cohort and their family members.

Total cost savings for the seven patients that reached Delphi consensus and were modeled was $184,846. This included $156,575 in hospital cost savings (length of stay, avoided procedures) and $28,271 in avoided professional fees. To arrive at that total, we also subtracted incurred costs, specifically the cost of the additional administered doses of IVIG and associated physician fees for patient 6180 and the cost of earlier administration of Factor XIII replacement and associated professional fees for patient 6207 as a result of the molecular diagnoses. We compared the total costs of sequencing the cohort of 38 patients and their families with the costs saved in the seven cases modeled. Total cost of sequencing the patients and families was $239,400. Total cost savings were therefore negative $54,554, indicating a modest net financial loss (Figure 1). A total of 12.1 QALYs were gained in two of the seven patients modeled (Figure 1). Most of these gains were due to the difference in quality of life attributed to a decreased risk of mortality and neurologic complication for patient 6207. The difference in total costs (cost savings minus costs of sequencing) for the 38 probands and families, as stated above, was $54,554 (net negative). Thus, we spent $54,554 to save 12.1 QALYs, a cost of $4,509 per QALY. Interventions with an ICER below $50,000 are considered high value (22–24, 30, 31).

## Discussion

Genetic disorders are a leading cause of mortality in the NICU and PICU, and affected children are disproportionally admitted to intensive care units (32–37). Rapid whole genomic sequencing for diagnosis of monogenic disorders is becoming increasingly applicable to critical care, as evidence continues to demonstrate that timely deployment of precision medicine leads to optimal clinical care (1, 2, 38–41). As with any new technologic advancement, the benefits of rWGS must be systematically evaluated while considering costs, societal acceptability, and willingness of funders to reimburse. In the United States especially, where healthcare costs are higher per capita than in any other nation (42), information regarding cost-effectiveness may be especially important for health system financiers and policymakers. Despite increasing evidence that sequencing improves clinical outcomes and more recent data demonstrating that it also reduces costs of care, routine application of rWGS in the ICU has remained a challenge, perhaps largely in part due to exceedingly rare reimbursement for testing by payers (1, 38, 43–47). Much of the cost-effectiveness data initially emerged from countries with universal health care and its application to a fragmented United States healthcare system could be complex (12, 16, 17, 48, 49). Farnaes et al. analyzed a cohort of six NICU patients in the United States diagnosed by rWGS and showed a reduction in hospital length of stay by 124 days resulting in a combined inpatient cost savings of $803,200 (approximately $19,000 per infant) (4). Subsequently, in 2018, the California state legislature commissioned and funded Project Baby Bear to determine if the economic and clinical benefits of rWGS could be reproduced at five different sites across the state. In this quality improvement project, Dimmock et al. completed an examination of 184 prospectively-evaluated critically ill children under 1 year of age who received rWGS across five ICUs in California (9). A diagnostic rate of 40% in this cohort, coupled with a 32% change in clinical management for diagnosed patients, produced a net cost savings of between $500,000 and $1.2 million ($12,041 to $15,786 per infant sequenced, in USD) (9). While results for both of these investigations demonstrated that rWGS can be implemented cost-effectively for critically ill infants in the United States, the children in these two cohorts were less than one year old, leaving open the question of whether these findings translate to children in the PICU up to 18 years of age. In this study, we present the first cost utility analysis of rWGS in the PICU in the United States to our knowledge.

We were able to quantify the impact in healthcare utilization for seven of 13 (54%) previously published PICU cases in which a molecular diagnosis was made by rWGS and a resultant change in management occurred. rWGS was estimated to lead to a reduction in the cost of care for these seven patients by $184,846 after subtracting incurred clinical costs. These estimates are conservative and reflect the judgment of independent Delphi panels. Most of the cost savings was due to reductions in the length of hospital stay. Conclusive diagnoses enabled clinicians to prognosticate illness trajectory more confidently; in some cases (patients 6147 and 6193), this information empowered parents to shift their goals for clinical care to comfort rather than curative or prolonged care. After accounting for the cost of sequencing all 38 patients in the PICU cohort, as well as their families ($239,400), there was a net financial loss of $54,554. However, the consensus of the Delphi panels was that rWGS diagnoses also resulted in a gain of 12.1 QALYs, averaging a cost of $4,509 per QALY. Even the most conservative analysts regard an intervention with cost-per-QALY under $20,000 to be justifiable, while the most commonly used willingness-to-pay thresholds by funders are higher, ranging from $50,000-100,000 per QALY or higher (22–24, 30, 31). Therefore, despite a small net financial loss, when taking the QALY gains into account the analysis of this PICU cohort provides robust cost-effective evidence in favor of rWGS in PICUs.

There are several limitations in this study. Although also appearing to reduce rather than increase total costs, the impact of precision medicine could not be quantified in six of the 13 diagnosed patients with changes in management attributable to first line rWGS (five patients in whom changes could not be financially modeled (Supplementary Table 1) and one patient, 6052, in whom Delphi did not reach consensus). The number of patients modeled limits our generalizability. We also did not systematically obtain the costs of tests or other investigations that were avoided due to performing rWGS. In a related study, costs of avoided testing averaged $162-$378 per patient (9). Additionally, this is a small study with a heterogeneous population, limiting generalizability and introducing potential bias. Due to the rarity of these diseases and the novelty of some of the molecular diagnoses identified, we were unable to use historical matched controls and instead relied upon counterfactual trajectories that were confirmed (or refuted) by an independent Delphi panel. This approach (counterfactual trajectory with expert panel consensus) was chosen because we knew from experience that the children receiving rWGS diagnoses would have rare genetic diseases, making identification of matched historical controls unlikely (50, 51). Furthermore, insufficient information exists in the literature regarding natural history or routine clinical practice for many of these uncommon disorders (4, 38, 41). Some assumptions, supported by the available literature, were made about long-term patient outcomes. We also included incurred costs as a result of rWGS diagnosis (patients 6180 and 6207). These patients were diagnosed between 2016 and 2018 and we reported the costs at that time and have not adjusted for inflation. Our conservative approach may significantly underestimate the actual benefits accrued. The IRB for the original cohort study (5) did not allow for reporting of variants of uncertain significance (VUS), which possibly could have led to incurred costs if VUS results compelled clinicians to order additional testing. Generalization of this data to the broader United States context assumes that the costs for a given medication or procedure at Rady Children's Hospital are representative of other facilities. Clinical management may also vary based on institution, as will decisions to proceed with palliative care. The analysis assumed that average charges over the last three inpatient days of a patient's hospitalization were a fair substitute for the charges of an avoided inpatient day. Finally, this study is unlikely to be applicable outside of the US healthcare system, as costs of ICU treatment may vary widely between different countries.

The patients in our original cohort received rWGS early and quickly while in the PICU. Other studies have shown that early diagnosis results in a higher clinical benefit and cost savings (4, 9, 14, 52). It is likely that our cost utility analysis benefited from timely ascertainment of the underlying molecular diagnosis. Recently, faster turnaround times (TAT) (1, 2, 9) afford clinicians opportunities to avoid further investigations and affect management leading to cost savings and gained QALYs. If rWGS had been used later in the clinical course, these impactful windows for high-yield intervention may be missed. The current TAT in 2021 compared to this historical cohort also limits generalizability here (1, 2, 9). In sensitivity analyses of the Project Baby Bear study, Dimmock et al. found proportionately lower cost savings for TATs of 7 or 14 days compared to TATs of 3 days. The TAT for the seven patients who reached consensus and were analyzed in our cohort was 17.6 days (mean was 13.6 for the entire cohort of 38) (5). Thus, it is possible that if we had had more rapid TATs during the original study (2016-2018), potentially the cost savings could have been more substantial in this cohort. Making a genetic diagnosis has the potential to be beneficial at any point in life, but evidence suggests that identifying children early and quickly, prior to significant damage and definitive medical decision points, results in the greatest utility (9, 38).

Though this study focused particularly on cost analysis of rWGS in critically ill children, the economic factor is only one component of many that need to be considered when evaluating potential implementation of rWGS in the PICU. Future studies should also continue to explore the societal impact of implementing rWGS, including from the perspectives of families whose children have received rWGS. Surveys of these families in one rWGS NICU study found that that although only 23% of infants received an rWGS diagnosis, 97% of parents reported that genomic testing was at least somewhat useful (53). It would be important to determine whether or not PICU families feel similarly. Furthermore, the PICU tends to be more clinically heterogeneous than the NICU, owing in part to the wider age range, thus it may be beneficial to parse out which subsets of PICU patients (perhaps by clinical presentation, body system affected, age, etc.) are most likely to receive rWGS diagnoses that lead to changes in management and even changes in clinical outcome.

This study supports the use of rWGS for critically ill children with suspected single locus genetic conditions from an economic (including cost-per-QALY) perspective. Future studies should evaluate the comparative effectiveness of children with lower acuity and/or more broad use of rWGS as recent studies in infants have demonstrated cost effectiveness when routinely sequencing all children admitted to NICU without clear etiology (9). With such testing now approved by some commercial insurers (44) and anticipated state sponsored programs (54, 55), studies should be performed to better understand the barriers to broader implementation.

## Materials and Methods

### Study Design

This study was approved by the institutional review board (IRB) at the University of California, San Diego. Retrospective comparison of healthcare utilization following molecular diagnosis by rapid genome sequencing was evaluated for a previously described cohort of inpatients in the PICU at Rady Children's Hospital in San Diego, California. Written informed consent was obtained from at least one parent or guardian. Details of the original investigation design, workflow, and inclusion/exclusion criteria have been previously published (5). The present analysis was performed utilizing this same cohort of patients to determine the financial impact of rWGS in critically ill children.

### Selection of Affected Children

**Figure 2:**
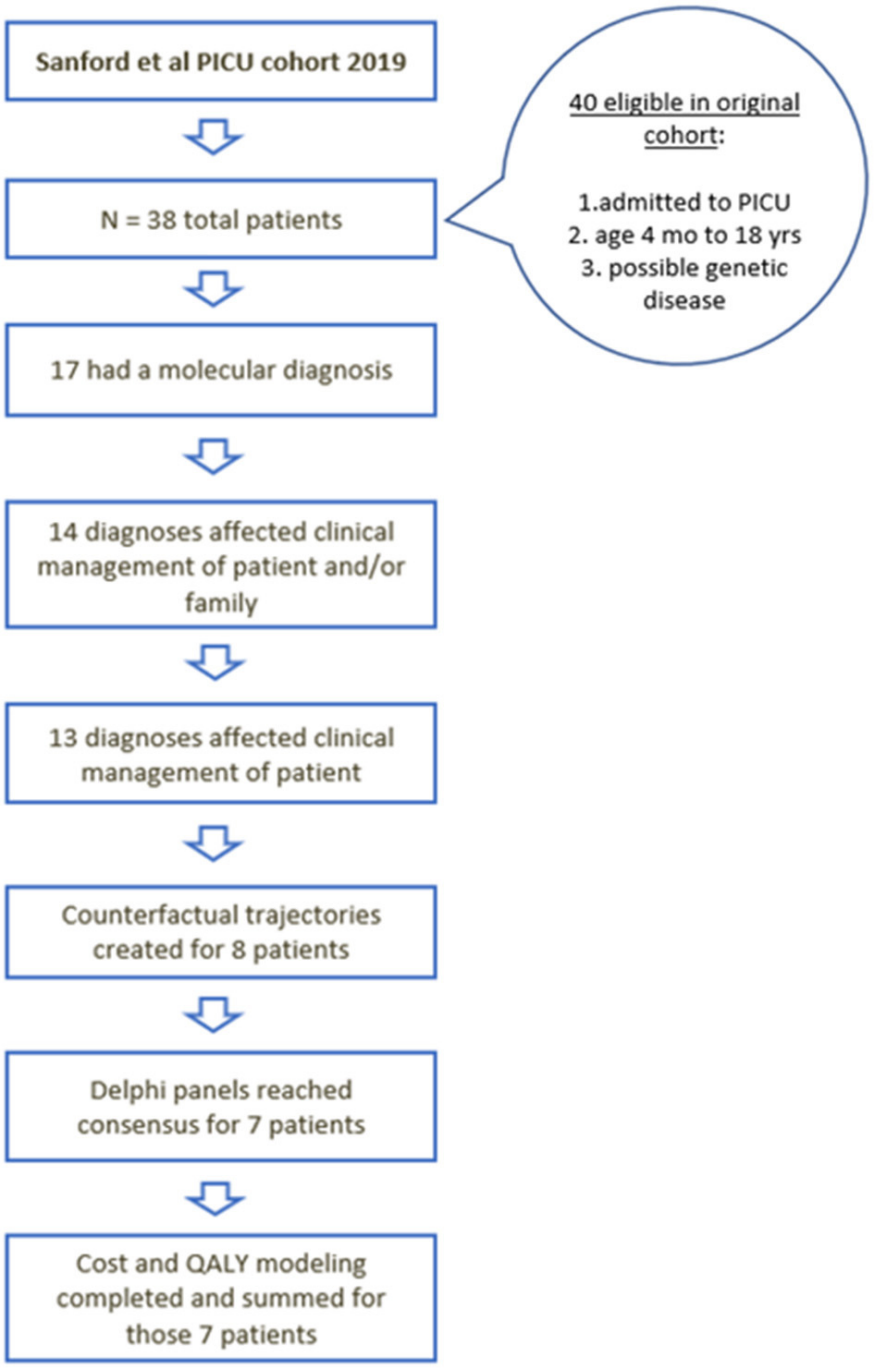
Flow diagram of the children who were initially included, received diagnosis, had changes in management, and underwent cost utility analysis.

Characteristics of the initial cohort of 38 children in the PICU who received rWGS have been described elsewhere (5). Seventeen of the 38 children received a molecular diagnosis by rWGS and 13 had a change in their clinical management as a result of the genomic diagnosis (Figure 2) (5). Of those 13, detailed QALY and ongoing cost of care modeling was modeled for eight patients (Table 1). The economic effect of precision medicine was unable to be quantitatively determined in the remaining five children (Supplementary Table 1).

### Sequencing and Bioinformatics

Analysis DNA was isolated, and 2x100 or 2x150 nucleotide rWGS was performed to ~45-fold coverage as previously described (4). Sequence alignment to the reference human genome and nucleotide variant calling was by DRAGEN (Illumina Inc, San Diego, CA) (4). An automated copy number variation pipeline was implemented in July 2017, which identified CNVs with a combination of the tools Manta and CNVnator (56, 57). Prior to July 2017, any CNV diagnoses were made via manual inspection of raw genomic data. Variants were annotated, analyzed, and interpreted with Opal Clinical (Fabric Genomics, Oakland, CA). All causative SNVs were confirmed by Sanger sequencing and CNVs were confirmed by MLPA and qPCR or aCGH. No variants failed confirmation.

### Phenotyping Information, Variant Filtering, and Variant Interpretation

Clinical features were manually extracted from electronic medical records (EMR) and translated into Human Phenotype Ontology (HPO) terms. The HPO terms were used in analysis in two ways: (1) To generate a patient-specific gene list (58–60) to provide an initial filtered list of variants; and (2) To prioritize variants by phenotypic overlap and potential variant consequence using VAAST and Phevor in the Opal Clinical platform (61). Nucleotide variants were filtered by predicted consequence, an allele frequency <0.5% in the ExAC and gnomAD databases, and inheritance pattern if parental data was available. Variants in genes previously implicated in human disease were required for clinical reporting. CNVs were filtered by events overlapping the OMIM Morbid gene list (~4,000 genes) and an internal frequency of <2%. Variants selected for full manual curation underwent gene curation to determine the strength of the known gene-disease relationship and the overlap with the patient's disease, and all variants were interpreted in accordance with the American College of Medical Genetics/Association of Molecular Pathology (ACMG/AMP) recommendations (62, 63). Per IRB protocol and initial guidance from the FDA, only pathogenic and likely pathogenic variants were allowed to be reported. Variants of uncertain significance could not be reported. We did not specifically evaluate for any of the 59 genes identified by the ACMG/AMP as reportable incidental findings (64). During the consent process, families were offered the choice to opt out of receiving medically actionable incidental findings that were inadvertently discovered.

### Delphi Panel

We utilized a modified Delphi method (25) to establish consensus for counterfactual trajectories including the anticipated standard of care in the absence of rWGS. The Delphi panel consisted of ten pediatric critical care board certified physicians at ten independent institutions. It is difficult to identify matched controls for patients suffer from rare genetic diseases and more often than not there is not enough disorder-specific information in published literature to dictate concrete clinical management practices for many of these rare diseases (4, 9, 38, 43). Consequently, we used a counterfactual trajectory with a modified Delphi panel to establish consensus for the counterfactual trajectories as previously described (4, 5). An external panel of ten attending pediatric intensivists board-certified in pediatric critical care from ten unique institutions was assembled. Panelists were not currently funded by RCIGM and did not have financial ties to RCIGM. Panelists did receive an honorarium of $200 for survey completion. For the final patient, 6207, because the patient had a rare (*n* = 133 in the literature) and specific hematologic genetic disorder that had the potential to significantly impact his quality of life and life expectancy, we considered that it was more appropriate for pediatric hematologists to evaluate the counterfactual trajectory. Thus, a panel of five pediatric hematologists from three different institutions was gathered.

The panel developed consensus on counterfactual trajectories using the Delphi Method, a structured, systematic method that uses consensus expert opinion to make an educated decision (4, 5, 25). Within the counterfactuals, when the molecular diagnosis was considered in the differential diagnosis, we utilized available literature on time to diagnosis and turnaround times for clinically available testing to form a reasonable assessment for when correct testing (gene panel, single gene sequencing, etc.) would have been obtained if rWGS had not been available. For ultra-rare or atypical presentations of syndromes this was difficult to assess. Consensus methods such as the Delphi are increasingly used in medicine to develop guidelines for controversial subjects and to make consensus-based determinations when insufficient information is available (51). Expert panelists review the available knowledge and are surveyed for their responses to specific questions. For this study, the questions posed to the Delphi panel were developed by the authors with guidance from medical geneticists and health economics experts. The replies are scored to determine the variation in response; if consensus is not reached, the questions are returned to the panelists for a second round, with the mean of responses from the previous round visible to the panelists, but the panelists remain anonymous to one another and the study team. The process is halted after a predefined stop criterion is reached; in this case, the criterion was the completion of two rounds. The questions that did not reach consensus after two rounds were excluded from the analysis.

The Likert scale was used as the survey instrument. The Likert items available to select from where: strongly disagree, disagree, neutral (neither agree nor disagree), agree, strongly agree, or unable to comment. Panelists had the option to indicate that they did not have sufficient expertise to answer a question (unable to comment), and their score was then excluded for that question. The answers were given numerical scores: strongly disagree was scored as 1, disagree as 2, neutral as 3, agree as 4, and strongly agree as 5. The mean consensus score for each question was then calculated. Questions with a mean consensus score of >/= 4 (agree/strongly agree) or < /=2 (disagree/strongly disagree) were considered to reach consensus. Questions that did not meet consensus during the first round were marked with the group's score and were returned to the panelists for a second round, so that each panelist was aware of the group mean when they responded to the questions in the second round. If the panelists failed to reach consensus on a question in the second round, those items were discarded. Likert scale scoring by individual panelists can be found in Supplementary Table 5. Only the replies that met consensus were included in cost modeling.

### Cost Modeling

Healthcare utilization was modeled in eight of 13 patients in whom rWGS resulted in a change in clinical management that could be quantified by comparing actual healthcare utilization with that of a counterfactual diagnostic trajectory as has been previously described (4, 9). A modified Delphi method was used to establish consensus for counterfactual trajectories as described above (4, 5, 25). Data obtained included both cost savings and costs incurred due to molecular diagnosis. To determine the costs for these specific patients, billing personnel from Rady Children's Hospital extracted all associated clinical investigations, interventions, procedures, medications, and inpatient room costs from the medical records. Counterfactual resource utilization was estimated based on values available in the literature and institutional costs. Professional costs were estimated by multiplying the accrued hospital costs by a published ratio for professional services (65). Modeling and methods for estimating associated costs are further detailed in the Supplementary Materials.

### QALY Modeling and Cost Utility Analysis

Quality of life (QOL) was modeled using QALYs. The formula used was QALY = QOL ^*^ number of years. QOL adjustments used for each case are shown in Supplementary Table 3. Estimates of QOL adjustments for mild, moderate, and severe neurological devastation and death, as well as life expectancy for each of these cases, were obtained from literature review (22, 26–29, 66–69). Life expectancy was modified based on gender. Cost per QALY is the preferred assessment tool used by payers to optimize resource allocation, with each QALY gained valued in the United States at ~$50,000–$100,000 (21–24). Cost effectiveness of rapid GS was measured with the incremental cost effectiveness ratio (ICER). The ICER is the cost per incremental gain in quality adjusted life years.

### Estimating Cost Savings From Reduced Length of Stay and Avoided Procedures

For patients with identified increases in length of stay or procedures we intended to capture the actual costs of these days or procedures. For patients with an anticipated change in the length of stay as determined by Delphi consensus, the average cost of the patients last 3 days of hospitalization without procedures were utilized to estimate the daily cost of hospitalization. For avoided procedural costs, one to three comparable cases were identified and included anesthesia, operating room, and supply charges.

## Data Availability Statement

The raw economic data supporting the conclusions of this article will be made available by the authors, without undue reservation. While no DNA sequence was generated as a part of this published work, all novel DNA sequence variants have been uploaded to ClinVar under our institutional identifier, Organization ID: 506081. For further details see Supplementary Table 6. All data associated with this study are present in the paper, Supplementary Materials, or are available at the Longitudinal Pediatric Data Resource under a data use agreement and subject to the limitations of the informed consent documents for each subject (Accession Number nbs000003.v1.p, https://nbstrn.org/tools/lpdr). Requests to access the datasets should be directed to the corresponding author (Erica Sanford Kobayashi).

## Ethics Statement

This study was approved by the Institutional Review Board (IRB) at the University of California, San Diego. Written informed consent to participate in this study was provided by the participants' legal guardian/next of kin. Written informed consent was obtained from the minor(s)' legal guardian/next of kin for the publication of any potentially identifiable images or data included in this article.

## Author Contributions

ES, NC, and DD: conceptualization. BW, BE, KP, EA, BB, CG, NR, JG, AD, EI, and CT: data curation. BW and WB: formal analysis and resources. SK: funding acquisition. LF, DD, SC, and BW: methodology. SK and CH: supervision. ES, DD, and NC: visualization. ES: writing—original draft. ES, NC, CH, SK, DD, BW, CT, AD, and SR: writing—review and editing. All authors contributed to the article and approved the submitted version.

## Funding

This study was supported in part by grant U19HD077693 from National Institute of Child Health and Human Development and National Human Genome Research Institute.

## Author Disclaimer

The views expressed in this article are those of the author(s) and do not necessarily reflect the official policy or position of the Department of the Navy, Department of Defense, or the United States Government.

## Conflict of Interest

BW is a salaried employee for Invitae with stock holdings. EA work has been funded by NIH T32 DK 104717-5. BB disclosed that he is employed by the U.S. Navy. SR is an employee of Exact Sciences Inc. and owns stock in the company. DD reports previous consulting fees from Audentes, Biomarin, Ichorion, and Complete Genomics. DD reports having a patent, Methods and Apparatus of Identification of Disease Associated Mutations, US8718950B2, licensed to HudsonAlpha Institute for Biotechnology. The remaining authors declare that the research was conducted in the absence of any commercial or financial relationships that could be construed as a potential conflict of interest.

## Publisher's Note

All claims expressed in this article are solely those of the authors and do not necessarily represent those of their affiliated organizations, or those of the publisher, the editors and the reviewers. Any product that may be evaluated in this article, or claim that may be made by its manufacturer, is not guaranteed or endorsed by the publisher.

## References

[1] Clark MM Hildreth A Batalov S Ding Y Chowdhury S Watkins K. Diagnosis of genetic diseases in seriously ill children by rapid whole-genome sequencing and automated phenotyping and interpretation. Sci Transl Med. (2019) 11:489. 10.1126/scitranslmed.aat6177 31019026 PMC9512059

[2] Owen MJ Niemi A-K Dimmock DP Speziale M Nespeca M Chau KK. Rapid sequencing-based diagnosis of thiamine metabolism dysfunction syndrome. N Engl J Med. (2021) 384:2159–61. 10.1056/NEJMc2100365 34077649 PMC9844116

[3] Willig LK Petrikin JE Smith LD Saunders CJ Thiffault I Miller NA. Whole-genome sequencing for identification of Mendelian disorders in critically ill infants: a retrospective analysis of diagnostic and clinical findings. Lancet Resp Med. (2015) 3:377–87. 10.1016/S2213-2600(15)00139-3 25937001 PMC4479194

[4] Farnaes L Hildreth A Sweeney NM Clark MM Chowdhury S Nahas S. Rapid whole-genome sequencing decreases infant morbidity and cost of hospitalization. NPJ Genom Med. (2018) 3:10. 10.1038/s41525-018-0049-4 29644095 PMC5884823

[5] Sanford EF Clark MM Farnaes L Williams MR Perry JC Ingulli EG. Rapid whole genome sequencing has clinical utility in children in the PICU. Pediatr Crit Care Med. (2019) 20:1007–20. 10.1097/PCC.0000000000002056 31246743 PMC6832787

[6] Clark MM Stark Z Farnaes L Tan TY White SM Dimmock D. Meta-analysis of the diagnostic and clinical utility of genome and exome sequencing and chromosomal microarray in children with suspected genetic diseases. NPJ Genom Med. (2018) 3:16. 10.1038/s41525-018-0053-8 30002876 PMC6037748

[7] Mestek-Boukhibar L Clement E Jones WD Drury S Ocaka L Gagunashvili A. Rapid Paediatric Sequencing (RaPS): comprehensive real-life workflow for rapid diagnosis of critically ill children. J Med Genet. (2018) 55:721–8. 10.1136/jmedgenet-2018-105396 30049826 PMC6252361

[8] Wu ET Hwu W-L Chien Y-H Hsu C Chen T-F Chen N-Q. Critical trio exome benefits in-time decision-making for pediatric patients with severe illnesses. Pediatr Crit Care Med. (2019) 20:1021–6. 10.1097/PCC.0000000000002068 31261230

[9] Dimmock D Caylor S Waldman B Benson W Ashburner C Carmichael JL. Project Baby Bear: rapid precision care incorporating rWGS in 5 California children's hospitals demonstrates improved clinical outcomes and reduced costs of care. Am J Hum Genet. (2021) 108:1–8. 10.1016/j.ajhg.2021.05.008 34089648 PMC8322922

[10] Tan TY Dillon OJ Stark Z Schofield D Alam K Shrestha R. Diagnostic impact and cost-effectiveness of whole-exome sequencing for ambulant children with suspected monogenic conditions. JAMA Pediatr. (2017) 171:855–62. 10.1001/jamapediatrics.2017.1755 28759686 PMC5710405

[11] Stark Z Tan T-Y Chong B Brett GR Yap P Walsh M. A prospective evaluation of whole-exome sequencing as a first-tier molecular test in infants with suspected monogenic disorders. Genet Med. (2016) 18:1090–6. 10.1038/gim.2016.1 26938784

[12] Stark Z Schofield D Alam K Wilson W Mupfeki N Macciocca I. Prospective comparison of the costeffectiveness of clinical whole-exome sequencing with that of usual care overwhelmingly supports early use and reimbursement. Genet Med. (2017) 19:867–74. 10.1038/gim.2016.221 28125081

[13] Stark Z Lunke S Brett GR Tan NB Stapleton R Kumble S. Meeting the challenges of implementing rapid genomic testing in acute pediatric care. Genet Med. (2018) 20:1554–63. 10.1038/gim.2018.37 29543227

[14] Schofield D Rynehart L Shresthra R White Stark SM Z. Long-term economic impacts of exome sequencing for suspected monogenic disorders: diagnosis, management, reproductive outcomes. Genet Med. (2019) 21:2586–93. 10.1038/s41436-019-0534-x 31110331

[15] Lacaze P Tiller J Winship I. Healthcare system-funded preventive genomic screening: challenges for australia and other single-payer systems. Public Health Genomics. (2019) 22:140–4.. 10.1159/000502917 31550728

[16] Stark Z Schofield D Martyn M Rynehart L Shrestha R Alam K. Does genomic sequencing early in the diagnostic trajectory make a difference? A follow-up study of clinical outcomes and cost-effectiveness. Genet Med. (2019) 21:173–80. 10.1038/s41436-018-0006-8 29765138

[17] Chung CCY Leung GKC Mak CCY Fung JLF Lee M Pei SLC. Rapid whole-exome sequencing facilitates precision medicine in paediatric rare disease patients and reduces healthcare costs. The Lancet Regoinal Health- Western Pacific. (2020) 14:55. 10.1016/j.lanwpc.2020.100001 34327338 PMC8315561

[18] Grosse SD Farnaes L. Genomic sequencing in acutely ill infants: what will it take to demonstrate clinical value? Genet Med. (2019) 21:269–71. 10.1038/s41436-018-0124-3 30100610 PMC6691721

[19] Bobinac A van Exel NJ Rutten FF Brouwer WB. Valuing QALY gains by applying a societal perspective. Health Econ. (2013) 22:1272–81. 10.1002/hec.2879 23080321

[20] Weinstein MC Torrance G McGuire A. QALYs: the basics. Value Health. (2019) 12:S5–9. 10.1111/j.1524-4733.2009.00515.x 19250132

[21] Bao EL Chao L-Y Ni P Moura LMVR Cole AJ Cash SS. Antiepileptic drug treatment after an unprovoked first seizure: A decision analysis. Neurology. (2018) 91:e1429–39. 10.1212/WNL.0000000000006319 30209239 PMC6177278

[22] Neumann PJ Cohen Weinstein JT MC. Updating cost-effectiveness–the curious resilience of the $50,000-per-QALY threshold. N Engl J Med. (2014) 371:796–7. 10.1056/NEJMp1405158 25162885

[23] Bobinac A van Exel J Rutten Brouwer FF WB. The value of a QALY: individual willingness to pay for health gains under risk. Pharmacoeconomics. (2014) 32:75–86. 10.1007/s40273-013-0110-1 24293198

[24] Grosse SD. Assessing cost-effectiveness in healthcare: history of the $50,000 per QALY threshold. Expert Rev Pharmacoecon Outcomes Res. (2008) 8:165–78. 10.1586/14737167.8.2.165 20528406

[25] Helmer-Hirschberg O. Analysis of the future: the delphi method. In Santa Monica, CA: RAND Corporation. (1967).

[26] Naderi M Zarei T Haghpanah S Eshghi P Miri-Moghaddam E Karimi M. Intracranial hemorrhage pattern in the patients with factor XIII deficiency. Ann Hematol. (2014) 93:693–7. 10.1007/s00277-013-1918-7 24149912

[27] Naderi M Alizadeh S Kazemi A Tabibian S Zaker F Bamedi T. Central nervous system bleeding in pediatric patients with factor XIII deficiency: a study on 23 new cases. Hematology. (2015) 20:112–8. 10.1179/1607845414Y.0000000172 25001244

[28] Dorgalaleh A Naderi M Shamsizadeh M. Morbidity and mortality in a large number of Iranian patients with severe congenital factor XIII deficiency. Ann Hematol. (2016) 95:451–5. 10.1007/s00277-015-2568-8 26692088

[29] Siboni SM Zanon E Sottilotta G Consonni D Castaman G Mikovic D. Central nervous system bleeding in patients with rare bleeding disorders. Haemophilia. (2012) 18:34–8. 10.1111/j.1365-2516.2011.02545.x 21539694

[30] Huang L Frijters P Dalziel K Clarke P. Life satisfaction, QALYs, and the monetary value of health. Soc Sci Med. (2018) 211:131–6. 10.1016/j.socscimed.2018.06.009 29935403

[31] Ryen Svensson LM. The willingness to pay for a quality adjusted life year: a review of the empirical literature. Health Econ. (2015) 24:1289–301. 10.1002/hec.3085 25070495

[32] March March of Dimes Foundation Data Book for Policy Makers: Maternal Infant and Child Health in the United States 2016. Available online at: http://www.marchofdimes.org/March-of-Dimes-2016-Databook.pdf (accessed January 20, 2020).

[33] Berry MA Shah PS Brouillette RT Hellmann J. Predictors of mortality and length of stay for neonates admitted to children's hospital neonatal intensive care units. J Perinatol. (2008) 28:297–302. 10.1038/sj.jp.7211904 18046336

[34] Kochanek KD Murphy SL Xu J Arias E : Mortality in the United States, 2016. NCHS Data Brief. (2017) 293:1–8. Available online at: https://www.cdc.gov/nchs/products/databriefs/db293.htm 29319473

[35] Weiner J Sharma J Lantos J Kilbride H. How infants die in the neonatal intensive care unit: Trends from 1999 through 2008. Arch Pediatr Adolesc Med. (2011) 165:630–4. 10.1001/archpediatrics.2011.102 21727274

[36] Hagen Hansen CM TW. Deaths in a neonatal intensive care unit: A 10-year perspective. Pediatr Crit Care Med. (2004) 5:463–8. 10.1097/01.PCC.0000128893.23327.C1 15329163

[37] Stevenson DA Carey JC. Contribution of malformations and genetic disorders to mortality in a children's hospital. Am J Med Genet. (2004) 126:393–7. 10.1002/ajmg.a.20409 15098237

[38] Kingsmore SF Cakici J Clark MM Gaughran M Feddock M Batalov S. A randomized, controlled trial of the analytic and diagnostic performance of singleton and trio, rapid genome and exome sequencing in ill infants. Am J Hum Genet. (2019) 105:719–33. 10.1016/j.ajhg.2019.08.009 31564432 PMC6817534

[39] Bainbridge MN Wiszniewski W Murdock DR Friedman J Gonzaga-Jauregui C Newsham I. Whole-genome sequencing for optimized patient management. Sci Transl Med. (2011) 3:87re3. 10.1126/scitranslmed.3002243 21677200 PMC3314311

[40] Soden SE Saunders CJ Willig LK Farrow E Smith LD Petrikin JE. Effectiveness of exome and genome sequencing guided by acuity of illness for diagnosis of neurodevelopmental disorders. Sci Transl Med. (2014) 6:265ra168. 10.1126/scitranslmed.3010076 25473036 PMC4286868

[41] Stavropoulos DJ Merico D Jobling R Bowdin S Monfared N Thiruvahindrapuram B. Whole-genome sequencing expands diagnostic utility and improves clinical management in paediatric medicine. NPJ Genom Med. (2016) 1:15012. 28567303 10.1038/npjgenmed.2015.12 PMC5447450

[42] Organisation for Economic Co-operation and Development 2019. Available online at: https://data.oecd.org/healthres/health-spending.htm (accessed January 15, 2021).

[43] Petrikin JE Cakici J Clark MM Willig LK Sweeney NM Farrow EG. The NSIGHT1-randomized controlled trial: rapid whole-genome sequencing for accelerated etiologic diagnosis in critically ill infants. NPJ Genom Med. (2018) 3:6. 10.1038/s41525-018-0045-8 29449963 PMC5807510

[44] California BSo. Whole Exome and Whole Genome Sequencing for Diagnosis of Genetic Disorders. 2020. Available online at: https://www.blueshieldca.com/bsca/bsc/public/common/PortalComponents/provider/StreamDocumentServlet?fileName=PRV_WholeExome_Sequen.pdf (accessed August 20, 2020).

[45] Bick D Fraser PC Gutzeit MF Harris JM Hambuch TM Helbling DC. Successful Application of Whole Genome Sequencing in a Medical Genetics Clinic. J Pediatr Genet. (2017) 6:61–76. 10.1055/s-0036-1593968 28496993 PMC5423809

[46] French CE Delon I Dolling H Sanchis-Juan A Shamardina O Megy K. Whole genome sequencing reveals that genetic conditions are frequent in intensively ill children. Intensive Care Med. (2019) 45:627–36. 10.1007/s00134-019-05552-x 30847515 PMC6483967

[47] Wang H Lu Y Dong X Lu G Cheng G Qian Y. Optimized trio genome sequencing (OTGS) as a first-tier genetic test in critically ill infants: practice in China. Hum Genet. (2020) 139:473–82. 10.1007/s00439-019-02103-8 31965297

[48] Hayeems RZ Bhawra J Tsiplova K Meyn MS Monfared N Bowdin S. Care and cost consequences of pediatric whole genome sequencing compared to chromosome microarray. Eur J Hum Genet. (2017) 25:1303–12. 10.1038/s41431-017-0020-3 29158552 PMC5865210

[49] Australian Genomics Health Alliance 2021. Available online at: https://www.australiangenomics.org.au/ (accessed February 1, 2021).

[50] Yousuf MI. Using experts' opinions through Delphi technique. Pract Assess Res Eval. (2007) 12. 10.7275/rrph-t210

[51] Pearson I Rothwell B Olaye A Knight C. Economic modeling considerations for rare diseases. Value Health. (2018) 21:515–24. 10.1016/j.jval.2018.02.008 29753347

[52] Meng L Pammi M Saronwala A Magoulas P Ghazi AR Vetrini F. Use of exome sequencing for infants in intensive care units: ascertainment of severe single-gene disorders and effect on medical management. JAMA Pediatr. (2017) 171:e173438. 10.1001/jamapediatrics.2017.3438 28973083 PMC6359927

[53] Cakici JA Dimmock DP Caylor SA Gaughran M Clarke C Triplett C. A Prospective Study of Parental Perceptions of Rapid Whole-Genome and -Exome Sequencing among Seriously Ill Infants. Am J Hum Genet. (2020) 107:953–62. 10.1016/j.ajhg.2020.10.004 33157008 PMC7675003

[54] Michigan Department of Health Human Services Notice of Proposed Policy Available online at: https://www.michigan.gov/documents/mdhhs/2131-Lab-P_728798_7.pdf (accessed July 10, 2021).

[55] California, AB-114 Medi-cal benefits: rapid whole genome sequencing https://leginfo.legislature.ca.gov/faces/billTextClient.xhtml?bill_id=202120220AB114. (accessed July 10, 2021).

[56] Chen X Schulz-Trieglaff O Shaw R Barnes B Schlesinger F Kallberg M. Manta: rapid detection of structural variants and indels for germline and cancer sequencing applications. Bioinformatics. (2016) 32:1220–2. 10.1093/bioinformatics/btv710 26647377

[57] Abyzov A Urban AE Snyder M Gerstein M. CNVnator: an approach to discover, genotype, and characterize typical and atypical CNVs from family and population genome sequencing. Genome Res. (2011) 21:974–984. 10.1101/gr.114876.110 21324876 PMC3106330

[58] Köhler S Schulz MH Krawitz P Bauer S Dolken S Ott CE. Clinical diagnostics in human genetics with semantic similarity searches in ontologies. Am J Hum Genet. (2009) 85:457–64. 10.1016/j.ajhg.2009.09.003 19800049 PMC2756558

[59] Köhler S Vasilevsky NA Engelstad M Foster E McMurry J Ayme S. The Human Phenotype Ontology in 2017. Nucleic Acids Res. (2016). 10.1093/nar/gkw1039 PMC5210535 27899602

[60] Yang H Robinson PN Wang K. Phenolyzer: phenotype-based prioritization of candidate genes for human diseases. Nat Methods. (2015) 12:841–3. 10.1038/nmeth.3484 26192085 PMC4718403

[61] Singleton MV Guthery SL Voelkerding KV Chen K Kennedy B Margraf RL. Phevor combines multiple biomedical ontologies for accurate identification of disease-causing alleles in single individuals and small nuclear families. Am J Hum Genet. (2014) 94:599–610. 10.1016/j.ajhg.2014.03.010 24702956 PMC3980410

[62] Richards S Aziz N Bale S Bick D Das S Gastier-Foster J. Standards and guidelines for the interpretation of sequence variants: a joint consensus recommendation of the American College of Medical Genetics and Genomics and the Association for Molecular Pathology. Genetics in Medicine. (2015) 17:405–24. 10.1038/gim.2015.30 25741868 PMC4544753

[63] Coonrod EM Margraf RL Russell A Voelkerding Reese KV MG. Clinical analysis of genome next-generation sequencing data using the Omicia platform. Expert Rev Mol Diagn. (2013) 13:529–40. 10.1586/14737159.2013.811907 23895124 PMC3828661

[64] Kalia SS Adelman K Bale SJ Chung WK Eng C Evans JP. Recommendations for reporting of secondary findings in clinical exome and genome sequencing, 2016 update (ACMG SF v2.0): a policy statement of the American College of Medical Genetics and Genomics. Genet Med. (2017) 19:249–55. 10.1038/gim.2017.17 27854360

[65] Peterson C Xu L Florence C Grosse Annest SD JL. Professional Fee Ratios for US Hospital Discharge Data. Med Care. (2015) 53:840–9. 10.1097/MLR.0000000000000410 26340662 PMC4681390

[66] Husebye ES Anderson Kämpe MS O. Autoimmune Polyendocrine Syndromes. N Engl J Med. (2018) 378:1132–41. 10.1056/NEJMra1713301 29562162 PMC6007870

[67] Ferre EM Rose SR Rosenzweig SD Burbelo PD Romito KR Niemela JE. Redefined clinical features and diagnostic criteria in autoimmune polyendocrinopathy-candidiasis-ectodermal dystrophy. JCI Insight. (2016) 1:e88782. 10.1172/jci.insight.88782 27588307 PMC5004733

[68] Foss Abrahamsen A Hoiby EA Hannisdal E Jorgensen OG Holte H Hasseltvedt V. Systemic pneumococcal disease after staging splenectomy for Hodgkin's disease 1969-1980 without pneumococcal vaccine protection: a follow-up study 1994. Eur J Haematol. (1997) 58:73–7. 10.1111/j.1600-0609.1997.tb00927.x 9111586

[69] Chong J Jones P Spelmanm D Leder Cheng K AC. Overwhelming post-splenectomy sepsis in patients with asplenia and hyposplenia: a retrospective cohort study. Epidemiol Infect. (2017) 145:397–400. 10.1017/S0950268816002405 27776576 PMC9507625

