# Supplementary material for: Cost Efficacy of Rapid Whole Genome Sequencing in the Pediatric Intensive Care Unit
Source: Front Pediatr. 2022 Jan 24;9:809536. doi: 10.3389/fped.2021.809536 (PMC8818891; doi:10.3389/fped.2021.809536)
Supplement: Supplementary file 1 [file Data_Sheet_1.pdf]

Supplementary Material

Cost efficacy of rapid whole genome sequencing in the pediatric intensive care unit

Supplementary Data……………………………………………………………………Page 2

Supplemental References……………… ……………………………………………...….Page 10

Supplemental Table S1:…..…………… ……………………………………………….Page 12

Supplemental Table S2:………… …………………….………………………………….Page 13

Supplemental Table S3:…………………… …………………………………………...…Page 14

Supplemental Table S4:……………… ………………………………………………...Page 15

Supplemental Table S5:..………… ……………………………………………………….Page 16

Supplemental Table S6:..………… ……………………………………………………….Page 17

Supplementary Data

Counterfactual trajectories, Delphi questions, and Panel Consensus

Patient 6007 was a 14 month-old female with a history of intractable complex partial epilepsy that had failed phenobarbital and levetiracetam, and at the time of admission to the general pediatric ward for status epilepticus she was taking topiramate and clobazam. During the admission, lacosamide was started, which improved but did not resolve the seizures. The neurologist's next plan was to either add rufinamide or start the patient on a continuous midazolam infusion, but before these options were initiated, rWGS identified an 11.4Mb deletion that included *PCHD19*. Case reports suggested a short course of steroids might improve seizure clustering, most often immediately after the initial administration in females with *PCDH19* variants.[1,2] With this knowledge, methylprednisolone 30mg/kg x 3 days was administered with subsequent seizure abatement, and the patient was discharged home on topiramate, lacosamide, clobazam, and a steroid taper.

The *PCHD19* variant was reported on a microarray that resulted two weeks later. It is unlikely that the planned addition of rufinamide would have led to seizure abatement as previously reported there was only a 17% response rate.[3] In one study of PCDH19 patients, efficacy of continuous administration of midazolam in suppressing the ongoing seizure clusters was confirmed in 13 of 17 patients.[4] It is likely seizures would have stopped with a midazolam infusion, but this has the potential for increased escalation medical management including respiratory and hemodynamic support of which the steroid treatment did not. In one study of pediatric patients receiving continuous midazolam infusion for convulsive status epilepticus, 17 patients (32%) required mechanical ventilation.[5] The literature suggests that the average duration of midazolam therapy when used specifically to abate pediatric status epilepticus is 47.5 hours[6], though this is not specific to children with *PCDH19* variants.

Delphi panel questions :

1. The rWGS diagnosis led to the use of pulse methylprednisolone in this patient. (strongly agree, agree, neutral, disagree, strongly disagree, unable to comment) Consensus: Agree
2. The rWGS diagnosis prevented admission to the PICU for a midazolam infusion. (strongly agree, agree, neutral, disagree, strongly disagree, unable to comment) Consensus: Agree
3. The minimum anticipated ICU stay would be two days. (strongly agree, agree, neutral, disagree, strongly disagree, unable to comment) Consensus: Agree
4. If started on a continuous midazolam infusion, the patient would have had approximately a 1 in 3 chance of requiring mechanical ventilation[5] (strongly agree, agree, neutral, disagree, strongly disagree, unable to comment) Consensus: Agree

Patient 6052 was a 3-year-old female with a history of global developmental delay and complex partial seizures, who presented with lactic acidosis, encephalopathy, diminished cardiac function, and rhabdomyolysis. She was found to be compound heterozygous for variants in *TANGO2*, a disorder that causes recurrent metabolic encephalomyopathic events and associated neurodegeneration. As a result of this diagnosis, she was started on ubiquinol, B50, L-carnitine, and vitamin B2 as a variety of cofactors have been used as supplements in mitochondrial depletion syndromes with some evidence of stabilization in laboratory values and functional testing.[7] The metabolic attending physician in this case documented clinical improvement concomitant with initiation of cofactor treatment in this patient. The patient's parents were also provided with an

emergency letter describing her diagnosis and treatment. She has already been to the ER once since her diagnosis during an acute encephalopathic episode and clinically improved after administration of the recommended high dextrose-containing intravenous fluids. A previous Delphi panel agreed that having an emergency letter from the metabolic physician facilitated the provision of appropriate medical care for this patient's uncommon disorder.[8]

Case series describing individuals afflicted with *TANGO2* demonstrate a propensity towards metabolic crises, which include rhabdomyolysis, hypoglycemia, and cardiac arrhythmias.[9] Data has shown that 10 out of 12 subjects with *TANGO2* presented with acute rhabdomyolysis.[10] Recurrent rhabdomyolysis can be fatal in severe cases, with an associated mortality rate of 8%–10%.[10] Furthermore, risk of acute renal failure in rhabdomyolysis is 5%.[11] Documented hypoglycemia occurred in 9 of 14 individuals during acute metabolic crisis or severe illness .[9] Electrocardiogram abnormalities occurred in 6 of 14 individuals in the setting of metabolic crisis, including prolonged QTc, torsades de pointes, ventricular tachycardia and/or fibrillation, and cardiac arrest (seen in 3 of 14 patients).[9]

Currently there are no published data to show efficacy in improving outcomes by use of mitochondrial cofactor supplementation in patients with *TANGO2*. However, the impression of the treating metabolic physician was that the patient was improving after initiation of this medication regimen. The admission in which she was diagnosed by rWGS was a total of eleven days. She has had one subsequent admission following her diagnosis, which was for only one day and during which the patient was appropriately treated with guidance from the metabolic team without developing any of the aforementioned medical complications typically associated with a metabolic crisis. As of March 2019, only 29 patients with *TANGO2* variants were described in the literature, as such, it is not yet possible to make significant inferences regarding length of hospitalization and frequency of crises specific to this disorder.[9] The limited published literature that does exist suggests that patients with pathogenic *TANGO2* variants can have prolonged hospitalizations of up to 4 weeks.[12] However, it may be meaningful to examine data from the parent group of mitochondrial disorders to which *TANGO2* belongs. In one study of 495 patients with mitochondrial disease, pediatric patients were hospitalized on average 1.1 times per year with median length of stay of 4 days per hospitalization event.[13]

Delphi panel question:

1. The molecular diagnosis likely reduced the length of subsequent hospital stays from four to eleven days to one day, with an average of 1.1 hospitalizations per year. (strongly agree, agree, neutral, disagree, strongly disagree, unable to comment) No consensus reached

Patient 6147 was a 4 month-old previously healthy infant admitted with severe anemia, elevated transaminases, and failure to thrive found to have homozygous pathogenic variants in *TRNT1*. *TRNT1* variants cause a clinical syndrome of sideroblastic anemia with B-cell immunodeficiency, periodic fevers, and developmental delay (OMIM #616084). Metabolic geneticists predicted a poor prognosis and advised palliative care consultation. The patient's mother initially expressed frustration at the limited treatment options explicitly stated that "even if it might not help" she planned to pursue full treatment. However, one month after molecular diagnosis her goals of care began to shift towards comfort care and avoiding hospitalizations even at the cost of prolonging life. In the subsequent months, she expressed understanding that there is no cure for his genetic condition, and began to view ongoing appointments, labs, and interventions to be futile and causing

the patient and the family symptoms not consistent with their goals of care. At eight months of age, his code status was changed to Do Not Attempt Resuscitation/Allow Natural Death and transitioned to hospice. A previous Delphi panel agreed that the molecular diagnosis facilitated this family's transition to palliative care.[8] When the patient was ten months old, he presented to the emergency room (ER) in critical condition with profound hypoglycemia and electrolyte derangements, and his family declined hospitalization. The patient was discharged from the emergency department within the same day. The mother was subsequently contacted via phone call within the next 24 hours due to a positive blood culture obtained during the ER visit. The blood culture was initially positive for coagulase negative Staphylococcus (CoNS) and additional molecular testing demonstrated methicillin resistant Staphylococcus epidermidis, a known culprit of sepsis especially in those who are immune-suppressed or who have indwelling medical devices and in particular this patient population.[14,15] For patients with demonstration of CoNS bacteremia, appropriate duration of treatment is at least five days and may be as long as 28 days depending on the manifestation of other signs and symptoms (Holland et al 2018 PMID 30264119).[16] Median pediatric ICU length of stay for sepsis is approximately 5 days and median hospital length of stay for sepsis is approximately 15 days.[17] Despite the blood culture result, the patient's mother chose not to bring the patient to the hospital for treatment and he died at home in the following days. In the absence of knowing the genetic diagnosis, the patient's mother would likely have perceived shifting away from life-prolonging measures as "giving up" without the counterbalancing measure of the expected poor outcome. The diagnosis allowed her to evaluate her goals and treatment preferences over time in the context of being unable to affect the expected poor outcome. Understanding the prognosis associated with the patient's *TRNT1* variant also allowed physicians to give the family latitude in the care plan, tailoring the plan according to mother's goals rather than strict medical criteria. Specifically, during different periods of acute illness, laboratory studies, endoscopy, a skin biopsy, and interventions based on results of echocardiogram were deferred because of understanding of prognosis and mom's goals of care accordingly. Prior to the final ER visit before his death, the patient's mother had discussions with palliative care and social worker about decreasing the number of outpatient subspecialty clinic appointments. Emergency department providers were ultimately comfortable with his discharge to home with hospice in a relatively guarded state due to their understanding of his diagnosis, prognosis, and family's goals of care.

Delphi panel questions:

1. The rWGS diagnosis avoided at least one hospitalization for sepsis (strongly agree, agree, neutral, disagree, strongly disagree, unable to comment) <u>Consensus</u>: Agree
2. Admission for sepsis would have resulted in an average ICU length of stay of 5 days and an average hospital stay of 15 days. (strongly agree, agree, neutral, disagree, strongly disagree, unable to comment) <u>Consensus</u>: Agree
3. The molecular diagnosis led to the family declining further interventions including a skin biopsy and EGD/intestinal biopsies. (strongly agree, agree, neutral, disagree, strongly disagree, unable to comment) <u>Consensus</u>: Agree

Patient 6153 was a 5 year-old female with prolonged QT syndrome who had recently been started on nadolol and was admitted to the PICU following a seizure-like event with subsequent laboratory work up that revealed severe hypocalcemia, hyperphosphatemia, and hypoparathyroidism. She was treated with intravenous calcium gluconate, magnesium sulfate, and teriparatide (recombinant

PTH) before being transitioned to enteral calcitriol and calcium carbonate. Repeat ECG following correction of the hypocalcemia demonstrated normalization of the QTc interval (433 ms), and therefore beta blockade was discontinued. rWGS identified compound heterozygous variants in *AIRE* (autoimmune regulator) gene in the proband which is a paternally inherited, pathogenic frameshift variant (NM_000383.3:c.1265del; p.Pro422LeufsTer58) and maternally inherited, likely pathogenic, missense variant (NM_000383.3:c.268T>C; p.Tyr90His). At the time of presentation, the patient did not knowingly meet the clinical criteria for autoimmune polyglandular syndrome type 1 (APS-1) as the diagnosis requires two out of three of the clinical features including chronic mucocutaneous candidiasis, hypoparathyroidism, and adrenal insufficiency.[18] Retrospectively, through chart review she did have a remote history of candidiasis, but her parents denied any memory of that condition. Because of this history, it is unlikely that gene sequencing for *AIRE* would have been sent early for this patient. The average age for development of a diagnostic dyad is 7.4 years and 12.1 years for the classic triad.[18] APS-1 is associated with a substantial risk for development of autoimmune adrenal insufficiency, with an incidence of 25-90%.[19] Once adrenal autoantibodies are present, the rate of development of adrenal insufficiency is 19% per year.[20,21] As a result of this diagnosis, the patient is undergoing regular surveillance with her endocrinologist for the development of adrenal insufficiency. Published recommendations for follow-up suggest that patients have a minimum of two visits per year with an endocrinologist with surveillance for 21-hydroxylase autoantibodies, laboratory evaluation of renal function, liver function, thyroid hormones, renin/renin activity, and hemoglobin related to other APS-1 disease manifestations.[22] Additionally, vaccination against pneumococcus (23-valent vaccine) and meningococcus is recommended, as patients can insidiously develop asplenia.[22] In one prospective observational study, asplenism developed in 8.6% of patients[18] and mortality from pneumococcal sepsis in unvaccinated asplenic patients in a prevaccine-era study of 325 patients was found to be 2.4%.[23] In a large registry of 3274 patients in the post-vaccine era, the incidence of invasive pneumococcal disease (mortality not reported) was 16/3274 (0.49%).[24] Therefore, a vaccinated patient with APS-1 would have an estimated reduction in their risk of death from pneumococcal sepsis of 0.16%.

Delphi panel questions:

1. The rWGS diagnosis resulted in regular surveillance for adrenal insufficiency and other APS-1 disease manifestations at least 2.4 years earlier than would have occurred using clinical criteria. (strongly agree, agree, neutral, disagree, strongly disagree, unable to comment) Consensus: Agree
2. Regular surveillance for adrenal insufficiency lead to semi-annual endocrinology clinic appointments, testing for 21-hydroxylase autoantibodies, and laboratory evaluation of renal function, liver function, thyroid hormones, renin/renin activity, and hemoglobin. (strongly agree, agree, neutral, disagree, strongly disagree, unable to comment) Consensus: Agree
3. Molecular diagnosis leading to vaccination for encapsulated organisms would decrease this patient's risk of dying from invasive pneumococcal disease by approximately 0.16% (strongly agree, agree, neutral, disagree, strongly disagree, unable to comment) Consensus: Agree

Patient 6159 was a 5 year-old previously healthy female admitted to the PICU for hypovolemic shock, respiratory failure requiring initiation of BiPAP, and acute kidney injury. During her hospitalization she developed ascites, peritonitis, pleural effusions, a pericardial effusion, anemia,

hepatitis, nephritis, and coagulopathy. There was no identified unifying diagnosis for her symptoms. Her acute glomerulonephritis was concerning for the possibility of an immune complex glomerulonephritis and a renal biopsy was recommended by the consulting nephrologist. However, rWGS revealed a heterozygous likely pathogenic NM_000092.4:c.4715C>T (p.Pro1572Leu) variant in the *COL4A4* gene. Defects in *COL4A4* have been associated with Alport syndrome and autosomal dominant thin basement membrane nephropathy.[25,26] Consensus guidelines recommend that patients with heterozygous *COL4A4* variants be managed as thin basement membrane nephropathy (TBMN)[26] as progression to end stage renal disease (ESRD) have been observed.[27] Consensus recommendation is that this particular group of patients be assessed for poor prognostic indicators (proteinuria, renal impairment, hypertension) and if identified, management by a nephrologist, potential initiation of an ACE inhibitor, and annual to biennial evaluation for hypertension, proteinuria, and renal impairment.[26] She was considered to carry a diagnosis of Alport syndrome by nephrology due to the molecular diagnosis and managed conservatively for thin basement membrane nephropathy, a renal biopsy was not performed.

Delphi panel question:

1. The molecular diagnosis avoided the need for a kidney biopsy in this patient. (strongly agree, agree, neutral, disagree, strongly disagree, unable to comment) <u>Consensus</u>: Agree

Patient 6180 was a previously healthy 19 month-old male admitted with pseudomonal septic shock. An immunodeficiency was suspected and lymphocyte subset analyses suggested a lack of B cells as an etiology, but sequencing the *BTK* gene to confirm X-linked agammaglobulinemia (XLA) typically takes four to six weeks. rWGS identified a *de novo*, pathogenic frameshift variant in *BTK* (NM_000061.2:c.726dup; ;p.Ile243TyrfsTer15) in four days. Prior to molecular diagnosis, the consulting immunologist recommended obtaining IgG levels every seven days and replacing for a level less than 400 mg/dL. With knowledge of the *BTK* variant, the recommendation was to obtain levels every 24 to 48 hours during the period of severe sepsis and administer IVIG if under 800 mg/dL. This resulted in ten administrations of IVIG over the first four weeks of PICU admission.

Delphi panel question:

1. The rWGS diagnosis leads to the administration of at least 6 extra/additional doses of IVIG over and above the number of doses of IVIG that would have been given without the molecular diagnosis. (strongly agree, agree, neutral, disagree, strongly disagree, unable to comment) <u>Consensus</u>: Agree

Patient 6193 was a 22 month-old female with a history of distal arthrogryposis and developmental delay who was admitted to the PICU after a cyanotic episode at home. Her parents reported prior apneic spells during sleep, such that they slept next to her so they could periodically stimulate her breathing. In the hospital, evaluation revealed both central and obstructive apnea. Bi-level positive pressure ventilation (BiPAP) was initiated during sleep with improvement. Without an explanation for the disordered breathing, her parents were reluctant to make decisions about goals of care. rWGS revealed a *de novo*, likely pathogenic missense variant in *NALCN* (NM_052867.3:c.1799A>G; p.Asp600Gly), which causes a syndrome of Congenital Contractures of the Limbs, Hypotonia, and Developmental Delay (CLIFAHDD; OMIM #616266) as well as respiratory insufficiency in some patients. This syndrome was recently described in 2015 through

analysis of a cohort of patient with arthrogryposis[28] and patients with *NALCN* variants have sleep-disordered breathing causing central apneas.[29] In another cohort of patients with variants in *NALCN*, two siblings were reported to have recurrent episodes of abnormal respiratory rhythm. Problems of respiration have also been established in a mouse model with deletions in *NALCN* leading to severely disrupted respiratory pattern and death within 24 hours of birth.[30] The molecular diagnosis facilitated engagement of palliative care and the transition to planning for home BiPAP prior to the patient leaving the ICU. In total, the patient was hospitalized for 26 days, 20 of which were in the PICU. Providing long-term ventilatory support for a variety of conditions associated with ventilatory failure in children is associated with an increase in survival and a decrease in the number of PICU admissions.[31,32] In one study of fifteen pediatric patients with neuromuscular disease of various etiologies, the initiation of non-invasive positive pressure ventilation (NPPV) resulted in patients spending 85% fewer days in the hospital (mean pre-NPPV 48.0 days, mean post-NPPV 7.0 days) and 68% fewer days in the PICU (mean pre-NPPV 12.0 days, mean post-NPPV 3.9 days) over the course of a year, with both findings reaching statistical significance.[32]

Delphi panel questions:

1. The rWGS diagnosis facilitated the transition to planning for home NPPV (as opposed to awaiting symptom resolution). (strongly agree, agree, neutral, disagree, strongly disagree, unable to comment) Consensus: Agree
2. The initiation of NPPV for this chronic neuromuscular condition would be expected to result in a decrease of hospital days and PICU days of approximately 85% and 68%, respectively, based on available data.[32] For this patient in particular, that is estimated to be 22 fewer hospital days on subsequent admission and 13.6 fewer PICU days. (strongly agree, agree, neutral, disagree, strongly disagree, unable to comment) Consensus: Agree

Patient 6207 was an 8 month-old previously healthy male who presented to his pediatrician's office for right arm paralysis and was brought to the emergency department by air ambulance. He had fallen less than one foot in height one week prior. He did not have loss of consciousness, but had progressive somnolence starting the day after the fall. A computed tomography angiogram of the head with and without contrast revealed a left frontal parietal, intra-axial mass, measuring approximately 4.3 x 5 x 4.1 cm, which was concerning for hemorrhagic neoplasm. The patient's activated partial thromboplastin time (aPTT) was 35 seconds (reference range 24 -38 seconds) and prothrombin time (PT) was 13.5 seconds with an international normalized ratio of 1.1 (reference range 11.4 – 14.0 seconds and 0.9 – 1.2 respectively). The fibrinogen was 224 mg/dL (reference range 160-425 mg/dL) and platelet count was 375 thousand per µL (reference range 140-440 thousand per µL). Magnetic resonance imaging of the brain with and without contrast showed a 5 x 5.4 x 4.6 cm heterogeneous intra-axial left frontal lobe lesion with a fluid level with sheet-like contrast enhancement. The margins of the lesions were well-defined and revealed chronic hemosiderin deposition leading to the suspicion that this was a large intraparenchymal hematoma from a recent cavernoma bleeding event. The patient was taken to the operating room on his fifth hospital day and the lesion was removed. Histopathology revealed the lesion to be hematoma without evidence of vascular malformation. He received packed red blood cell transfusion for anemia but did not receive plasma or other hemostatic agent. The patient was discharged from the hospital after 13 days with follow-up with neurology, neurosurgery, primary care, physical therapy, and occupational therapy. Given the diagnostic challenge, prior to discharge a Cerebral

Cavernoma Malformation (CCM) genetic panel (analysis of *CCM2*, *KRIT1*, and *PDCD10*) was sent, in addition to rapid trio whole genome sequencing.

The CCM genetic panel was negative, but rWGS identified a novel homozygous variant *F13A1* NM_000129.3:c.1352_1353del (p.His451ArgfsTer29). Hematology was immediately consulted, and urgent outpatient evaluation was scheduled. A Factor XIII activity level was less than 4%, confirming the diagnosis of severe FXIII deficiency. The patient was then started on prophylaxis with coagulation FXIII A-subunit (Recombinant) [Tretten®, Novo Nordisk, NJ, USA] receiving the first dose on post-operative day 30. Two months later the patient had an approximately 2.5 foot fall onto wood floor when sleeping next to mother. There was no loss of consciousness and the patient was taken to the Emergency room where a dose of recombinant FXIII (rFXIII) was given. Head CT showed no new bleeding at that time. The patient has had no further bleeding complications since starting rFXIII. One year after presentation the patient has residual right arm weakness and a mixed receptive expressive language delay for which he is receiving physical, occupational, and speech therapy. A previous Delphi panel consisting of pediatric critical care physicians concluded that the diagnosis of FXIII deficiency was made earlier because of rWGS, led to earlier administration of FXIII A-subunit, and likely prevented additional bleeding episodes and further neurologic injury.[8]

Studies have shown that nearly 1 in 4 patients (20/76) with FXIII deficiency who have had a CNS bleed and are not receiving prophylaxis will experience another CNS bleed.[33,34,35] Studies have shown that 12% (18/145) of patients die from their CNS bleed[51] with the mean age of death being 1.5 ± 2 years.[34] Of the 95 patients in the medical literature for which age of CNS bleed was available, the mean age at time of CNS bleed was 4.5 years, with a standard deviation of 5.6 years and a median of 3.5 years. Additionally, 83% of CNS bleeds occurred at 6 years of age or younger.[33,34,36] Of patients who survive, 72.6% have neurologic complications (53.5% psychological, 13.8% developmental, 13.8% locomotor disability, 6.8% speech impairment, 5.1% aphasia, 3.5% hemiplegia, and 3.5% visual impairment).[37] His initial hospital stay was 13 days and studies have shown the average length of stay in 514 cases of intracerebral hemorrhage regardless of cause to be 9.6 days.[38]

Hematology Delphi panel questions:

Factor XIII deficiency is rare, and the literature is sparse regarding re-bleeding risk with relatively small studies. This specific patient had a significant intracranial hemorrhage after falling <1 foot at an age much lower than the mean (8 months versus 4.5 years), then had a second fall of 2.5 feet within 2 months for which he received Factor XIII afterwards (in addition to regular prophylaxis prior). We are asking you, in your professional expert opinion, what you believe the likelihood is that the molecular diagnosis and resulting administration of Factor XIII changed his outcome from this second fall.

1. Is it more likely than not that this patient's second fall would have resulted in an intracerebral hemorrhage. (strongly agree, agree, neutral, disagree, strongly disagree, unable to comment) <u>Consensus</u>: Agree
2. If the patient had an intracerebral bleed due to the second fall, this more likely than not would have been substantial enough to result in additional disability. (strongly agree, agree, neutral, disagree, strongly disagree, unable to comment) <u>Consensus</u>: Agree

3. If the patient had not been diagnosed with FXIII deficiency during the hospitalization for the first bleed, and if he was thus admitted with a second CNS bleed after fall #2, this second CNS bleed would more likely than not have prompted a more thorough traditional hematology workup leading to the FXIII diagnosis. (strongly agree, agree, neutral, disagree, strongly disagree, unable to comment) Consensus: Agree

Supplemental References

1. Higurashi, N. et al. Immediate suppression of seizure clusters by corticosteroids in PCDH19 female epilepsy. *Seizure*. 27, 1-5 (2015).
2. Bertani, G. et al. Steroids efficacy in the acute management of seizure clusters in one case of PCDH19 female epilepsy. *Seizure*. 32,45-6 (2015).
3. Lotte, J. et al. Effectiveness of antiepileptic therapy in patients with PCDH19 mutations. *Seizure*. 35, 106-10 (2016).
4. Higurashi, N. et al. PCDH19-related female-limited epilepsy: further details regarding early clinical features and therapeutic efficacy. *Epilepsy Res*. 106, 191-9 (2013).
5. Nishiyama, M. et al. Fosphenytoin vs. continuous midazolam for pediatric febrile status epilepticus. *Brain Dev*. 40, 884-890 (2018).
6. Tasker, R.C. et al. Pediatric Status Epilepticus Research Group. Refractory Status Epilepticus in Children: Intention to Treat With Continuous Infusions of Midazolam and Pentobarbital. *Pediatr Crit Care Med*. 17, 968-975 (2016).
7. Rodriguez, M.C. et al. Beneficial effects of creatine, CoQ10, and lipoic acid in mitochondrial disorders. *Muscle Nerve*. 35, 235-42 (2007).
8. Sanford E.F. et al. Rapid Whole Genome Sequencing Has Clinical Utility in Children in the PICU. *Pediatr Crit Care Med*. 20, 1007-1020 (2019).
9. Dines, J.N. et al. TANGO2: expanding the clinical phenotype and spectrum of pathogenic variants. *Genet Med*. 21, 1899 (2019).
10. Lalani S.R. et al. Recurrent Muscle Weakness with Rhabdomyolysis, Metabolic Crises, and Cardiac Arrhythmia Due to Bi-allelic TANGO2 Mutations. *Am J Hum Genet*. 98, 347-57 (2016).
11. Mannix, R., Tan, M.L., Wright, R., & Baskin, M. Acute pediatric rhabdomyolysis: causes and rates of renal failure. *Pediatrics*. 11, 2119-25 (2016).
12. Ricci F. et al. Multi-system disorder and severe recurrent rhabdomyolysis due to *TANGO2* mutations in a 3 year-old child. *Neuromuscular Disorders*. 27, s207 (2017).
13. McCormack, S.E. et al. Hospitalizations for mitochondrial disease across the lifespan in the U.S. *Mol Genet Metab*. 121, 119-126 (2017).
14. Nguyen, T.H., Park, M.D., & Otto, M. Host Response to Staphylococcus epidermidis Colonization and Infections. *Front Cell Infect Microbiol*. 7, 90 (2017).
15. Cheung, G.Y. & Otto, M. Understanding the significance of Staphylococcus epidermidis bacteremia in babies and children. *Curr Opin Infect Dis*. 23,208-16 (2010).
16. Holland, T.L. et al. Effect of Algorithm-Based Therapy vs Usual Care on Clinical Success and Serious Adverse Events in Patients with Staphylococcal Bacteremia: A Randomized Clinical Trial. *JAMA*. 320, 1249-1258 (2018).
17. Balamuth, F. et al. Pediatric severe sepsis in U.S. children's hospitals. *Pediatr Crit Care Med*. 15, 798-805 (2014).
18. Ferre, E.M. et al. Redefined clinical features and diagnostic criteria in autoimmune polyendocrinopathy-candidiasis-ectodermal dystrophy. *JCI Insight*. 1, e88782 (2016).
19. Guo, C.J., Leung, P.S.C., Zhang, W., Ma, X., & Gershwin, M.E. The immunobiology and clinical features of type 1 autoimmune polyglandular syndrome (APS-1). *Autoimmun Rev*. 17, 78-85 (2018).
20. Ahonen, P., Miettinen, A., & Perheentupa, J. Adrenal and steroidal cell antibodies in patients with autoimmune polyglandular disease type I and risk of adrenocortical and ovarian failure. *J Clin Endocrinol Metab*. 64, 494-500 (1987).

21. Söderbergh A. et al. Prevalence and clinical associations of 10 defined autoantibodies in autoimmune polyendocrine syndrome type I. *J Clin Endocrinol Metab*. 89, 557-62 (2004).
22. Husebye, E.S., Anderson, M.S., & Kämpe, O. Autoimmune Polyendocrine Syndromes. *N Engl J Med*. 378, 1132-1141 (2018).
23. Foss Abrahamsen, A. et al. Systemic pneumococcal disease after staging splenectomy for Hodgkin's disease 1969-1980 without pneumococcal vaccine protection: a follow-up study 1994. *Eur J Haematol*. 58, 73-7 (1997).
24. Chong, J., Jones, P., Spelman, D., Leder, K., & Cheng, A.C. Overwhelming post-splenectomy sepsis in patients with asplenia and hyposplenia: a retrospective cohort study. *Epidemiol Infect*. 145, 397-400 (2017).
25. Kashtan, C.E. Alport Syndrome. 2001 Aug 28 [Updated 2019 Feb 21]. In: Adam MP, Ardinger HH, Pagon RA, et al., editors. GeneReviews® [Internet]. Seattle (WA): University of Washington, Seattle; 1993-2021. Available from: https://www.ncbi.nlm.nih.gov/books/NBK1207/
26. Savige, J. et al. Expert guidelines for the management of Alport syndrome and thin basement membrane nephropathy. *J Am Soc Nephrol*. 24, 364-75 (2013).
27. Papazachariou, L. et al. Frequent COL4 mutations in familial microhematuria accompanied by later-onset Alport nephropathy due to focal segmental glomerulosclerosis. *Clin Genet*. 92, 517-527 (2017).
28. Chong, J.X. et al. De novo mutations in NALCN cause a syndrome characterized by congenital contractures of the limbs and face, hypotonia, and developmental delay. *Am J Hum Genet*. 96, 462-73 (2015).
29. Campbell, J. et al. Deciphering Developmental Disorders Study, Urquhart DS. NALCN Dysfunction as a Cause of Disordered Respiratory Rhythm With Central Apnea. *Pediatrics*. 141, S485-S490 (2018).
30. Lu, B. et al. The neuronal channel NALCN contributes resting sodium permeability and is required for normal respiratory rhythm. *Cell*. 129, 371-83 (2007).
31. Chatwin, M., Tan, H.L., Bush, A., Rosenthal, M., & Simonds, A.K. Long term non-invasive ventilation in children: impact on survival and transition to adult care. *PLoS One*. 10, e0125839 (2015).
32. Katz, S., Selvadurai, H., Keilty, K., Mitchell, M., & MacLusky, I. Outcome of non-invasive positive pressure ventilation in paediatric neuromuscular disease. *Arch Dis Child*. 89, 121-4 (2004).
33. Naderi, M. et al. Central nervous system bleeding in pediatric patients with factor XIII deficiency: a study on 23 new cases. *Hematology*. 20, 112-8 (2015).
34. Naderi, M. et al. Intracranial hemorrhage pattern in the patients with factor XIII deficiency. *Ann Hematol*. 93, 693-7 (2014).
35. Siboni, S.M. et al. Central nervous system bleeding in patients with rare bleeding disorders. *Haemophilia*. 18, 34-8 (2012).
36. Naderi, M. et al. Polymorphism of thrombin-activatable fibrinolysis inhibitor and risk of intracranial haemorrhage in factor XIII deficiency. *Haemophilia*. 20, e89-92 (2014).
37. Dorgalaleh, A., Naderi, M., & Shamsizadeh, M. Morbidity and mortality in a large number of Iranian patients with severe congenital factor XIII deficiency. *Ann Hematol*. 95, 451-5 (2016).
38. Perkins, E., Stephens, J., Xiang, H., & Lo, W. The cost of pediatric stroke acute care in the United States. *Stroke*. 40, 2820-7 (2009).

Supplemental Table S1: Changes in management unable to be financially modeled

Patient	Gene	Diagnosis Name	Medication Change	Surgery/Procedure Change	Imaging/Surveillance Change	Palliative Care Consult	Morbidity Avoided
6031	*RYR2*	Catecholaminergic polymorphic ventricular tachycardia (CPVT)	Flecainide started	Avoided delay in ICD implantation			Potential recurrence of CPVT
6118	*ACVRL1*	Hereditary hemorrhagic telangiectasia, type 2			Brain MRI recommended every 3 to 5 years		Potential early detection of AVMs
6183	*MECP2*	Rett syndrome/neonatal encephalopathy				Yes	
7002	*KCNQ1*	Long QT syndrome, type 1	Nadolol started				Potential decreased risk of cardiac arrhythmias
7039	*TBCD*	Progressive encephalopathy with brain atrophy and thin corpus callosum				Yes	

ICD = implantable cardioverter defibrillator; AVM = arteriovenous malformation

Supplemental Table S2. Avoided interventions resulting in saved or incurred costs

Avoided Item	# of Items	Avoided Cost
PICU Day	18.6	$123,448
Inpatient Hospital (non-PICU) Day	18.4	$44,579
Step down care: PICU to non-PICU day	2	$8,322
Skin Biopsy	1	$340
Endoscopy	1	$9,283
IVIG Administrations	6	- $(8,374)
Factor XIII Replacement	3 (350 units each)	-$(27,438)
Kidney Biopsy	1	$6,415
Hospital Costs		$156,575
Professional Fees		$28,271
**Total**		**$184,846**

PICU = pediatric intensive care unit; IVIG = intravenous immunoglobulin

Supplemental Table S3. QALY Modeling for seven children with changes in clinical management as a result of rWGS

QALY Adjustments for Intellectual/ Developmental Disability	Severity:	
	None	1.00
	Mild	0.96
	Moderate	0.90
	Severe	0.84
**Patient 6207**		
	Life Expectancy 8-month old Male Hispanic+	79.10
	Risk of Death if not diagnosed	0.12
	Chance of survival if not diagnosed	0.88
	Chance of worsening Neuro Damage if survival if not diagnosed	0.73
	QALY Actual (8 month life expectancy x Mild impairment)	75.70
	QALY lost from death (actual * risk of dying)	9.08
	QALY lost from Brain damaged (Chance of survival*(Chance of brain damage*(Mild Impairment - Moderate impairment))*Life Expectancy	2.90
	Total QALY Saved	11.98
**Patient 6153**		
	Life Expectancy for 5 year old Female Non-Hispanic White+	76.40
	Risk of Death if not diagnosed	0.00
	QALY lost from death (actual * risk of dying)	0.12
	Total QALY Saved	0.12
**Total QALY Saved for all cases**		**12.10**

QALY = quality adjusted life years; rWGS = rapid whole genome sequencing

+https://www.cdc.gov/nchs/data/nvsr/nvsr68/nvsr68_07-508.pdf

Supplemental Table S4: Costs of sequencing

	Trio	Duo	Proband Only	Total
Patients	24	4	10	38
Costs	$ 7,400	$ 5,700	$ 3,900	
Total	$ 177,600	$ 22,800	$ 39,000	**$ 239,400**

Supplemental Table S5: Likert scale scoring by individual Delphi panelists- results after second and final round

	Panelist*									
Question	A	B	C	D	E	F	G	H	I	J	Tallied result (nearest 0.1)
**6007**											
1	5	5	4	5	5	5	5	5	5	5	4.9
2	4	3	4	3	5	4	4	5	5	5	4.2
3	4	4	4	5	4	4	4	4	5	3	4.1
4	3	3	4	4	4	4	5	4	5	4	4
**6052**											
1	3	3	4	4	4	4	4	3	4	4	3.7
**6147**											
1	4	5	5	5	5	5	3	5	5	4	4.6
2	4	5	4	5	4	4	2	5	4	4	4.1
3	3	5	4	4	5	5	3	5	5	4	4.3
**6153**											
1	4	5	3	5	5	5	4	5	5	5	4.6
2	4	5	4	5	5	4	4	5	5	4	4.5
3	4	5	3	5	3	4	4	4	5	4	4.1
**6159**											
1	4	5	5	3	3	5	5	4	5	5	4.4
**6180**											
1	4	5	4	5	5	5	5	4	5	4	4.6
**6193**											
1	4	5	4	5	3	5	3	4	4	5	4.2
2	4	4	4	4	4	4	4	4	4	4	4
**6207**											
	Panelist**										
Question	K	L	M	N	O						Tallied result (nearest 0.1)
1	5	5	4	4	5						4.6
2	5	5	3	5	4						4.4
3	5	5	5	4	4						4.6

*where A-J represent 10 individual pediatric intensivists from 10 unique United States hospitals; **where K-O represent 5 individual pediatric hematologists from 3 unique United States hospitals; 1 = strongly disagree, 2 = disagree 3 = neutral, 4 = agree, 5 = strongly agree, U=unable to comment; 4 and above or 2 and below were considered to reach consensus

Supplemental Table S6. Variants and their associated SCV accession numbers in ClinVar

Gene	Variant	SCV Accession Number	RCIGM ACMG classification*
NALCN	c.1799A>G	SCV000996111.1	Likely pathogenic
TANGO2	c.605+1G>A	SCV000996055.1	Pathogenic
TRNT1	c.443C>T	SCV000996076.1	Pathogenic
AIRE	c.1265delC and c.269A>G	SCV000609490.1 and SCV000609491.1	Pathogenic and Likely Pathogenic
COL4A4	c.4715C>T	VCV000017409	Likely Pathogenic
BTK	c.726dupT	SCV000809043	Pathogenic
F13A1	c.1352_1353delAT	SCV000804842	Likely Pathogenic
PCDH19	11.4 Mb deletion	Confirmed by CMA/not submitted	Pathogenic
ACVRL1	c.1450C>T	SCV000996066.1	Pathogenic
MECP2	c.789dupC	SCV000996099.1	Pathogenic
KCNQ1	c.573_577delGCGCT	SCV000996087.1	Pathogenic
TBCD	c.1340C>T and c.967C>T	SCV000996127.1 and SCV000996129.1	Likely Pathogenic and Pathogenic

*reflects scoring at time of sequencing
